# Feasibility and Acceptability of an Online Conversation Intervention to Promote Social Connectedness and Prevent Cognitive Decline among Older Chinese Immigrants

**DOI:** 10.1002/alz70858_100950

**Published:** 2025-12-25

**Authors:** Kexin Yu, Amanda B Mar, Sabrina A Lin, Kevin Duff, Lisa C Silbert, Hiroko H Dodge

**Affiliations:** ^1^ University of Texas Southwestern Medical Center, Dallas, TX, USA; ^2^ NIA‐Layton Aging & Alzheimer's Disease Research Center, Portland, OR, USA; ^3^ Oregon Center for Aging & Technology (ORCATECH), Portland, OR, USA; ^4^ Oregon Health & Science University, Portland, OR, USA; ^5^ Lewis & Clark University, Portland, OR, USA; ^6^ Portland Veterans Affairs Medical Center, Portland, OR, USA; ^7^ NIA‐Layton Aging & Alzheimer's Disease Center, Portland, OR, USA; ^8^ Massachusetts General Hospital, Harvard Medical School, Boston, MA, USA

## Abstract

**Background:**

Social isolation is a risk factor for developing Alzheimer's Disease and Related Dementias (ADRD). Due to their limited English proficiency and difficulties adjusting to the social environment, older Chinese immigrants can experience social isolation. The study examined the feasibility and acceptability of a culturally adapted behavioral health intervention to promote social connectedness and prevent cognitive decline in this at‐risk group. The intervention was adapted from the Internet‐Based Conversational Clinical Trial (I‐CONECT, ClinicalTrials.gov Identifier: NCT02871921), which studied whether user‐friendly video chats could enhance cognitive functions among socially isolated older adults. Although I‐CONECT showed efficacy in improving cognition and social satisfaction, it was limited to predominantly non‐Hispanic White participants.

**Methods:**

The Chinese Conversation Engagement Co‐design and Pilot Test (C‐CONECT) Study adapted the I‐CONECT intervention to be more culturally appropriate for Chinese immigrants. Participants completed 30‐minutes long, four times/week conversation sessions with trained research staff for a 6‐week intervention period. Outcomes assessed at baseline and post‐intervention included measures of loneliness, social isolation, depressive symptoms, and cognitive function. Participants elaborated on their experiences in qualitative interviews.

**Results:**

Eight older Chinese immigrants (mean age 76, 75% female, 6‐18 years of education) enrolled in the study, and seven completed the intervention. On average, the participants lived in the US for 25.3 years (range 1‐53). Seven opted for chats in Mandarin Chinese and one in English. The chat completion rate was 90%. Lower post‐intervention depressive symptoms were reported in 62% of the sample. In the qualitative interviews, participants viewed the chat sessions as warm and non‐judgmental. The primary motivation for participation was to improve their memory.

**Conclusions:**

The feasibility and acceptability of the C‐CONECT intervention were supported by the high completion rate of online conversations and participants’ satisfaction. These results suggest that a randomized clinical trial to determine the efficacy of the C‐CONECT intervention on cognitive function can be successfully implemented in an older Chinese immigrant population. Conducting this cultural adaptation of an existing evidence‐based intervention model lays the foundation for expanding effective behavioral health interventions for preventing ADRD in underrepresented populations.

